# Hernia uterine inguinale associated with Mayer-Rokitansky-Küster-Hauser syndrome: Three case reports and literature review

**DOI:** 10.1097/MD.0000000000032802

**Published:** 2023-02-03

**Authors:** Yifei Dai, Chenglu Qin, Linling Zhu, Guangnan Luo

**Affiliations:** a Department of Gynecology, Hangzhou Women's Hosptial, Hangzhou, Zhejiang, China; b Department of Gynecology, The Third Affiliated Hospital of Shenzhen University, Luohu People Hospital, Shenzhen, Guangdong, China.

**Keywords:** hernia, inguinale, Mayer-Rokitansky-Küster-Hauser (MRKH) syndrome, Müllerian aplasia, uterus

## Abstract

Mayer-Rokitansky-Küster-Hauser syndrome (MRKH syndrome) present with genital inguinal hernia was rare and probably under reported, on account of lack in typical gynecological symptom. It should be regarded with care.Here 3 cases diagnosed at our institution with detailed clinical information were present, and the literature was reviewed to paint a comprehensive profile of hernia uterine inguinale associated with MRKH syndrome.

**Patient concerns:**

Case no. 1 was a 36-year-old female with recurrent dragalgia for 5 years. Left rudimentary uterus at the left groin area was revealed by sonography scan and confirmed by diagnostic laparoscopy.Case no. 2 was a 27-year-old woman diagnosed with MRKH syndrome and her MRI examination suggested a suspicious swelling measuring 2.0cm×2.0cm in left groin. The left nonfunctionally rudimentary uterus and adnexa were incarcerated in the left inguinal hernial sac, which was revealed by laparoscopy.Case no. 3 was a 29-year-old woman, admitted with right abdominal pain with a provisional diagnosis of appendicitis. After appendicectomy, pelvic exploration showed a part of left rudimentary uterus and elongated oviduct herniated through the left internal inguinal ring.

**Diagnoses:**

Hernia uterine inguinale associated with MRKH syndrome.

**Interventions and outcomes:**

Case no.1: When the rudimentary uterus was pulled out from the hernia sac, it appearance dark ocher. Then the left rudimentary uterus was removed and the indirect defect of inguinal duct was closed.The patient was followed up for 18 months with no recurrence of abdominal pain.Case no.2 and 3:The left rudimentary uterus were replaced from the hernia sac, and the indirect defect was fixed with sutures.The patients recovered smoothly without complications for 12-month follow-up.

**Lessons:**

Left involvement of rudimentary uterus was frequently observed in patients with MRKH syndrome, along with ipsilateral ovary and/or fallopian tube horned in the hernia. Abdominal pain or inguinale mass could be the chief complaints while some individuals were asymptomatic. Either surgical removal or replacement of rudimentary uterus was an effectively optional treatment strategy for hernia uterine inguinale.When a patient with MRKH syndrome presented with abdominal pain of unknown cause or inguinal mass, rudimentary uterine inguinal hernia should be suspected.

## 1. Introduction

Mayer-Rokitansky-Küster-Hauser (MRKH) syndrome is a kind of müllerian aplasia characterized by total or partial agenesis of the uterus and vagina, normal ovaries, and a normal female karyotype (46, XX), with a 1 in 5000 female births incidence. In patients with Mayer-Rokitansky-Küster-Hauser syndrome (MRKH syndrome), the estimated incidence of inguinal hernia is 6.4%, significantly higher than women in the general population.^[1]^ MRKH syndrome present with genital inguinal hernia was rare and probably under reported, on account of lack in typical gynecological symptom. It should be regarded with care.

Herein, we present 3 representative cases from our center and other 6 cases previously reported to perform a comprehensive literature review for inguinal hernia containing rudimentary uterus associated with MRKH syndrome.

## 2. Case report

### 2.1. Case no.1

**Figure 1.**
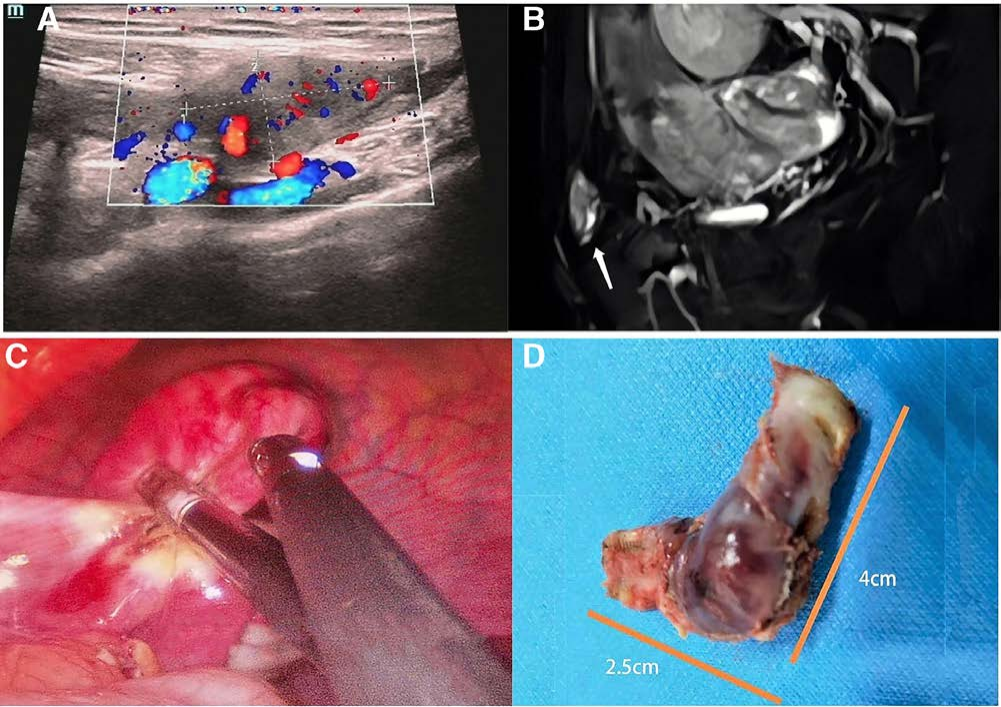
The imageology of case 1. (A) Sonography revealed a 2cm*2cm mass in the left groin area. Sagittal T2W MRI showed a mass in the left inguinal canal (white arrow). (C) Rudimentary uterus herniated through the internal inguinal ring. (D) The removed uterus. MRI = magnetic resonance imaging.

A 36-year-old woman complained of recurrent dragalgia at left groin for the past 5 years. The patient was lean built, and her secondary sexual characters were normal, as well as external genitalia. Ten years ago, she underwent laparoscopic-assisted vaginoplasty and right rudimentary hysterectomy (which had active endometrium inside) after she was diagnosed with MRKH syndrome. Laparoscopy showed an absent of left rudimentary uterus, accompanied by ovarian and oviducal dysplasia and adhesion to the left external inguinal canal. Over the longstanding course of the patient abdominal symptoms, she received most of the care at general surgery. Strangely, abdominal computed tomography (CT) scan and pelvic ultrasound showed no sign of inguinal mass. Conservative therapy with antibiotics achieved only minimal control of her symptoms. In 2021, she was readmitted for excruciating hypo gastralgia with an irreducibly inguinal swelling of 2.0cm × 2.0cm. The sonography scan was suspicious of left rudimentary uterus at the left groin area (Fig. 1A), which was confirmed by magnetic resonance imaging (MRI) (Fig. 1B). Subsequently, she underwent a diagnostic laparoscopy, revealing dysplastic ovaries and fallopian ducts on both sides. The left ovary was found to be adhered to a solid nodule at the left groin. After the left inguinal hernia sac was opened, a hidden rudimentary uterus was visible (Fig. 1C). Actually, the nodule exposed outside of the inguinal duct in pelvic was proved to be a part of the rudimentary uterus. When the rudimentary uterus was pulled out from the hernia sac, it was discovered that the uterine appearance was dark ocher (Fig. 1D). Then the left rudimentary uterus, with an overall size of 4.0cm × 2.5cm, was removed and the indirect defect of inguinal duct was closed. Clinic anatomization showed smooth muscle of the uterus without endometrial tissue. The patient was followed up for 18 months with no recurrence of abdominal pain.

### 2.2. Case no.2

**Figure 2.**
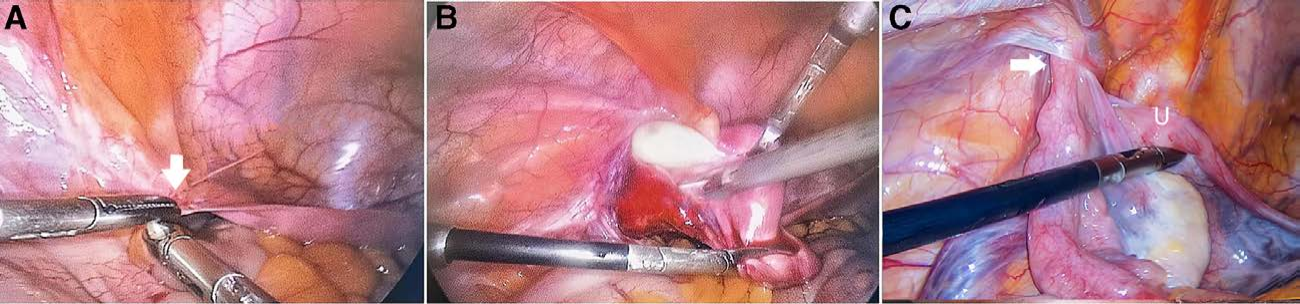
Laparoscopic image of case 2 and 3. (A) Left generative organ were absent. (B) Diagnostic hysteroscopy revealed the left rudimentary uterine and adnexa horned in the left inguinal canal. (C)In the left inguinal canal, a portion of the left rudimentary uterus and fallopian tube were found.

The patient is a 27-year-old woman diagnosed as MRKH syndrome, with primary amenorrhea. She had normal appearance of sexual characteristics. No evident abnormality showed in the hormonal profile and karyotype. Preoperative MRI suggested a suspicious swelling measuring 2.0cm × 2.0cm in left groin. A diagnostic laparoscopy revealed the left uterus were absent (Fig. 2A). The peritoneum formed a left inguinal hernial sac, which included left nonfunctionally rudimentary uterus and identical adnexa (Fig. 2B). The right rudimentary uterus, along with the right tube and ovary were intrapelvic. The left rudimentary uterus, ovary and tube were replaced from the hernia sac, and the indirect defect was fixed with sutures. The patient was followed up for 12 months with no complaint.

### 2.3. Case no.3

A 29-year-old female was admitted with right abdominal pain with a provisional diagnosis of appendicitis. Diagnostic laparoscopy demonstrated an inflamed appendix, followed by appendicectomy. Meanwhile, pelvic examination revealed a part of left rudimentary uterus and elongated oviduct herniating through the internal inguinal ring (Fig. 2C). Based on previous experience, the left rudimentary uterus, as well as the left tube, was replaced out of the dissected internal inguinal ring to the pelvic cavity. Finally, the surgeon shut off the indirect defect. Postoperative supplementary examination confirmed the patient clinical representations were consistent with MRKH syndrome. The patients recovered smoothly without complications for 12-month follow-up.

## 3. Methods

All relevant English literature was searched in internet databases, including PubMed and Web of Science, utilizing combinations of relevant medical subject heading terms, key words, and word variants for “Inguinal Hernia females,” “Uterus hernia,” “Hernia containing uterus,” and “Mayer-Rokitansky-Küster-Hauser,” “Mullerian duct abnormalities,” “Reproductive tract dysplasia.” Additional reports were found by manually searching reference lists of relevant papers and reviews. Reports without documenting the patient karyotype were excluded.

## 4. Discussion

The inguinal canal is a thin and diagonal tube in the lower anterior abdominal wall. Any intra-abdominal organ can herniate through inguinal canal and then present as inguinal hernia. Ovary is most common gynecologic organ herniated.^[1]^ Hernia uterine inguinale is extremely rare and its incidence significantly decreases along with the increasing of age,^[2]^ which is commonly found in women with maldevelopment of Müllerian duct.^[3]^ After reviewing all cases and published English literature, 6 cases were found about uterus contained in the hernia with MRKH. All cases were summarized in Table 1. Most of the patients were hospitalized with a groin swelling (4 cases were painful, 3 cases were painless), and the other 2 cases were asymptomatic. Hernia containing uterine tissue may also contain other Müllerian structures, including oviducts or ovaries.

**Table 1:** The cases of uterus contained in the hernia with MRKH syndrome.

No	Author/yr	Age(yr)	symptoms	Hernia site	Hernia contents	Treatment of rudimentary uterus	Hernia repair	Surgical approach	Comments
**1**	This article case 1	36	Swelling and pain in left groin	Left	Rudimentary uterus	Resection		Laparoscopy	-
**2**	This article case 2	27	Nontender left groin swelling	Left	Rudimentary uterus/ovary/oviduct	Replacement		Laparoscopy	
**3**	This article case 3	29	Asymptomatic	Left	Rudimentary uterus/ oviduct	Replacement		Laparoscopy	-
**4**	Al Omari W, 2011^[4]^	31	Nontender sliding groin swelling in right groin	Right	Rudimentary uterus/ovary/oviduct	Replacement	N/C	Laparotomy	Left kidney absent
**5**	Dadhwal V, 2011^[5]^	19	Abdominal pain; Nontender bilateral groin swelling	Bilateral	Rudimentary uterus	Resection	prolene mesh.	Laparoscopy	-
**6**	Kriplani A, 2000^[6]^	20	Asymptomatic	Left	Rudimentary uterine/ovary	Resection	N/c	Laparoscopy	Bilateral squint with microophthalmia
**7**	Jafari R, 2020^[3]^	13	Swelling and pain in left groin	Left	Rudimentary uterus/ovary/oviduct	Replacement	N/C	Laparotomy	Ectopic renal of left lower quadrant
**8**	Riggall FC, 1980^[7]^	16	Nontender left groin swelling	Left	Rudimentary uterus/ovary/oviduct	Resection	N/C	Laparotomy	-
**9**	Verma R, 2018^[8]^	45	A gradually increasing swelling in left groin with dragging pain	Left	Rudimentary uterus	Resection		Laparotomy	Left kidney absent

MRKH syndrome = Mayer-Rokitansky-Küster-Hauser syndrome.

Some scholars believe that herniation of malformed uterus can be attributed to the imperfect closure of congenital openings and failure of fusion of Müllerian ducts, which remains attached in the original position of the inguinal fold.^[9]^ On the other hand, abnormalities in the uterine suspensory ligaments may partly explain the presence of inguinal hernia.^[10]^ In case 4 and 6, surgeons observed a weak ligament of the uterus. Risk factors such as chronic cough or frequent heavy lifting, frequent valsava maneuvers may induce high intra-abdominal pressure and result in displacement of adnexal structures.^[11]^ This may be an aggravating factor for case 1 who works as a chiropractor.

Prodromidou A et.al^[12]^ state that involvement of the left side is much more frequent in cases of inguinal ovary hernia. Zeng K et.al^[13]^ hold the view that inguinal reproductive structures hernia are most commonly seen in the right side. In this study, hernia uterine inguinale is often located on the left side. So far, no theory is able to analyze the phenomenon and obtain a reasonable explanation. More samples are needed to clarify the statement in the future.

Abdominal pain or inguinale mass could be the chief complaints while some individuals were asymptomatic. The diagnosis of genital hernia should be taken into consideration, particularly among MRKH patients with lower abdominal pain. Its always inaccurate to perform ultrasound test alone before surgical operation. In case 7, the sonography showed the herniation sac containing ovarian-like structure and MRI showed left ovary, fallopian tube and rudimentary horned in left inguinal canal, which was confirmed by operation. In case 2, the genitals inguinal hernia was found by MRI before surgery, however, pelvic ultrasound showed no abnormalities. In case 4, the patient had a clinical diagnosis with right ovarian inguinal hernia by MRI. However, surgical exploration revealed an indirect hernia which contains a hypoplastic uterus, right ovary and fallopian tube. MRI, with an advantage in high resolution of soft tissue such as rudimentary uterus, is more recommended for preoperative examination.

Because of its commonly asymptomatic nature, the presence of uterus inguinal hernia associated with MRKH syndrome is unnoticeably and underreported. Contrast to the previous view that MRKH syndrome patients have no primordia uterus at all,^[14]^ we hypothesize that among some patients, the rudimentary uterus has migrated into the inguinal canal and fails to be identified by physical examination and imaging studies. As someone was diagnosed through surgery by accident (case 3 and case 6). Surgeons should explore the uterus and adnexa carefully and completely when either side of the reproductive organ is absent.

Most researchers believe ovarian incarceration increased the risk for ovarian torsion and infarction,^[2]^ therefore, early diagnosis and repositioning in such patients are important to protect the ovarian function. Due to the limited number of cases reported, there is no consensus surgical treatment for an inguinal hernia containing the uterus. All of the cases in Table1 underwent surgical exploration (laparoscopy for 5 cases and laparotomy for 4 cases). In 4 patients, the hernia contents (uterus with or without ovary or fallopian tube) were relocated, whereas in others, and excision of the uterus was undertaken. None of the resected uteri contained endometrial tissue. All patients recovered uneventfully without complications. For now, it appears that either removal or reduction of uterus is feasible. Although most MRKH syndrome uterus is infertile, some patients still express the demand for preservation of uterus. If there is no evidence for endometriosis or ischemia, either reposition or resection of the rudimentary uterus is practical. All aspects of care must be individualized according to the patient needs and the existing abnormalities.^[4]^

In conclusion, the evaluation and management of hernia uterine inguinale associated with MRKH syndrome must be holistic, especially with extensive counseling. Because it is often asymptomatic, a combination of pelvic ultra-sonography and MRI is recommended for patients with MRKH syndrome. The diagnosis of genital hernia should be taken into consideration in female with inguinal region mass, particularly among those who have lower abdominal pain. It is recommended to reposition the ovary contained in the sliding components carefully to protect reproductive endocrine function.

## Acknowledgments

The authors thank all the doctors, nurses in Shenzhen Luohu People Hospital of Gynecology for their assistance in our work.

## Author contributions

**Conceptualization:** Chenglu Qin, Guangnan Luo, Yifei Dai.

**Data curation:** Linling Zhu, Yifei Dai.

**Formal analysis:** Chenglu Qin, Yifei Dai.

**Methodology:** Guangnan Luo.

**Resources:** Linling Zhu, Yifei Dai.

**Supervision:** Chenglu Qin, Guangnan Luo.

**Writing – original draft:** Yifei Dai.

**Writing – review & editing:** Chenglu Qin, Guangnan Luo.
